# Putative determinants of virulence in *Melissococcus plutonius*, the bacterial agent causing European foulbrood in honey bees

**DOI:** 10.1080/21505594.2020.1768338

**Published:** 2020-05-26

**Authors:** Daniela Grossar, Verena Kilchenmann, Eva Forsgren, Jean-Daniel Charrière, Laurent Gauthier, Michel Chapuisat, Vincent Dietemann

**Affiliations:** a Department of Ecology and Evolution, Biophore, UNIL-Sorge, University of Lausanne, Lausanne, Switzerland; b Agroscope, Swiss Bee Research Centre, Bern, Switzerland; c Department of Ecology, Swedish University of Agricultural Sciences SLU, Uppsala, Sweden

**Keywords:** European foulbrood, EFB, *Melissococcus plutonius*, virulence, melissotoxin A, honey bee, *Apis mellifera*

## Abstract

Melissococcus plutonius

is a bacterial pathogen that causes epidemic outbreaks of European foulbrood (EFB) in honey bee populations. The pathogenicity of a bacterium depends on its virulence, and understanding the mechanisms influencing virulence may allow for improved disease control and containment. Using a standardized *in vitro* assay, we demonstrate that virulence varies greatly among sixteen *M. plutonius* isolates from five European countries. Additionally, we explore the causes of this variation. In this study, virulence was independent of the multilocus sequence type of the tested pathogen, and was not affected by experimental co-infection with *Paenibacillus alvei*, a bacterium often associated with EFB outbreaks. Virulence *in vitro* was correlated with the growth dynamics of *M. plutonius* isolates in artificial medium, and with the presence of a plasmid carrying a gene coding for the putative toxin melissotoxin A. Our results suggest that some *M. plutonius* strains showed an increased virulence due to the acquisition of a toxin-carrying mobile genetic element. We discuss whether strains with increased virulence play a role in recent EFB outbreaks.

## Introduction

The Western honey bee (*Apis mellifera* L.) is an insect of major worldwide ecological and economic importance. Honey bees produce honey and wax and pollinate many economically important crops[1]. The recent loss of managed honey bee colonies in several regions of the world threatens the ecological services provided by this pollinator [2–5]. Consequently, honey bee health has become a major concern not only for scientists, but also for the public and policy-makers [6–8]. Current research indicates that pathogens are a major cause of colony losses. Honey bee pathogens include viruses [9], protozoa [10], fungi [11], parasitic mites [12] and bacteria [7,9,13,14]. One of the most detrimental bacterial diseases affecting honey bees is European foulbrood (EFB). EFB is reported worldwide [15] and has emerged as an infectious disease since the mid-eighties in the United Kingdom, since the year 2000 in Switzerland and since 2010 in Norway [13,14,16,17]. High numbers of clinical cases have also been reported from Finland, France, Greece, Holland, Czechia and Italy [18–20], making EFB an economically important veterinary disease [18,21].

The pathogenic agent of EFB, *Melissococcus plutonius* (Lactobacillales, *Enterococcaceae*) [22], enters the intestinal tract of honey bee larvae through contaminated food provided by adult bees [23]. Once ingested, *M. plutonius* rapidly multiplies in the mid-gut lumen, possibly depriving the host of nutrients [24]. Diseased larvae typically change from white to a yellowish color, become flaccid and die 4–5 days after infection [23,25]. The massive loss of brood resulting from severe infection weakens the colony and can lead to its collapse [25].

To date, only broad-spectrum antibiotics such as oxytetracycline are available to treat EFB-affected colonies. Due to the risk of antibiotic resistance development [26,27] and an accumulation of residue in honey, the use of antibiotics is not a sustainable method to control EFB and is banned in some countries. In the absence of efficient treatment and given the severity of EFB outbreaks, 79 countries worldwide have classified EFB as a notifiable disease (World Animal Health Information Database, OIE [28]). In 22 of these countries, veterinary authorities destroy symptomatic colonies and monitor neighboring apiaries to avoid the further spread of the pathogen, which is costly and time-consuming. This situation calls for new control strategies, but their development is constrained by the limited knowledge of the pathogenesis of EFB [23].

Virulence is central to pathogenesis. A better knowledge of the mechanisms that determine this trait could contribute to the design of improved control methods. Indeed, virulence factors are promising targets for specific drugs or management measures [29]. Such measures include curing honey bee colonies hosting less virulent pathogens and restricting the use of destructive control methods to treat infections caused by more virulent pathogens [30]. An assessment of the extent and causes of virulence variation in strains of *M. plutonius* may thus prove useful in controlling EFB more efficient and more sustainable to improve honey bee health.

Early reports [31,32] suggested that *M. plutonius* isolates were genotypically and phenotypically homogeneous. As a result, variation in virulence was not expected. The discovery of genetic differences between isolates [33–40] has challenged this view. Differences in virulence have indeed been documented within *M. plutonius*, with strains defined as atypical by Arai et al.,2012 [34], killing a higher proportion of hosts in a shorter time period than typical strains. Differences in virulence also occur between typical strains, depending on experimental conditions [41,42]. However, no virulence factor has yet been clearly identified.

Several factors are likely to influence the virulence of pathogens. One is the impact of secondary agents [43]. In the case of EFB, saprophytic species such as *Paenibacillus alvei, Enterococcus faecalis, Brevibacillus laterosporus* or *Achromobacter eurydice* cause secondary bacterial infections that might increase damage to larvae [23,44]. However, their influence in EFB is debated [42,45–47]. Another potential cause of variation in virulence is the growth dynamics of the bacterial pathogen. Bacterial strains that multiply rapidly and reach high densities can cause more damage to the host [48,49]. Other major factors influencing virulence include the production of biologically active compounds, such as adhesins improving attachment to host cells, enzymes degrading host tissues, or toxins disturbing the physiological processes of the host [50–55].

Experiments aimed at quantifying the virulence of *M. plutonius* and identifying factors that influence virulence have been hampered by the complex social environment of the honey bee colony that affects the spread and growth of the bacteria [56,57], and by the legal obligation to destroy colonies showing disease symptoms [14]. These constraints can be overcome by experimentally infecting honey bee larvae reared *in vitro*. In the past, such assays were considered impossible due to *M. plutonius* loss of infectivity during bacterial sub-cultivation [46,56,58]. More recently, several studies using the *in vitro* larval rearing method [59] have circumvented this problem [34,41,42,47,60–62], making standardized quantification of virulence possible without any sanitary risk to the colonies in the field.

In this study, we reared honey bee larvae *in vitro* and infected them with *M. plutonius* to investigate the causes and extent of variation in virulence. We screened 17 *M. plutonius* isolates collected from five European countries for genetic differences and measured their virulence over the entire developmental period of worker brood. We examined whether virulence was associated with *M. plutonius* multilocus sequence type (MLST), co-infection with *P. alvei*, differences in growth dynamics in a culture medium and presence of a putative toxin-coding gene [39]. We discuss whether *M. plutonius* strains with high virulence play a role in recent EFB outbreaks and whether they could be targets for more sustainable control measures of the disease.

## Results

### *Virulence of* M. plutonius *strains*

**Figure 1.**
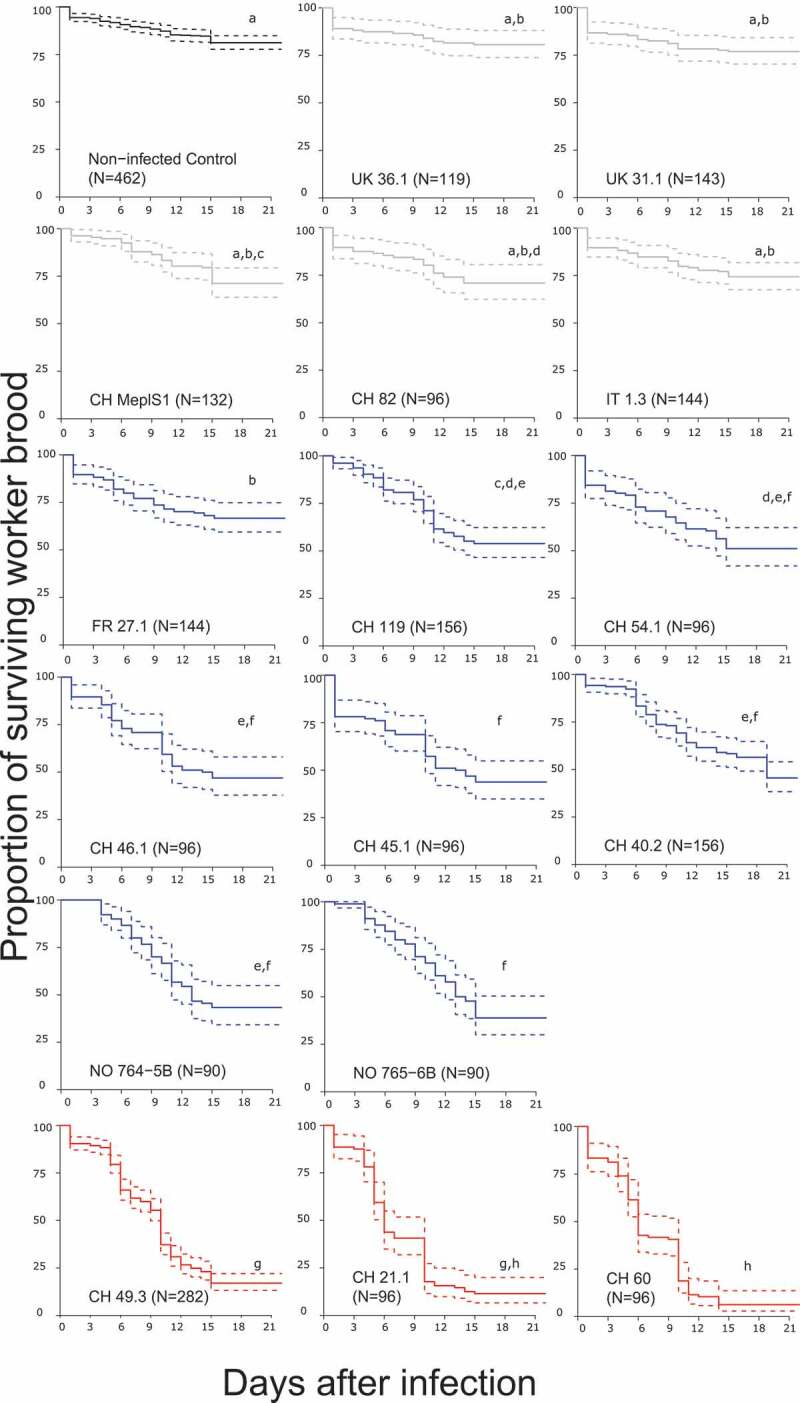
Survival of *in vitro* reared honey bee brood infected with 16 *M. plutonius* isolates (isolate code indicated in each panel). Grey, blue and red curves correspond to avirulent, low to intermediate and high virulence isolates, respectively. The black curve indicates survival of non-infected controls. N = number of larvae tested. Dashed lines represent 95% confidence intervals. Significant differences in the survival of brood due to treatments (uncorrected for control mortality) are indicated by different letters (pairwise log-rank tests, Bonferroni-Holm corrected, p < 0.001).

The *M. plutonius* isolates collected in various regions of Europe belonged to six multilocus sequence types and two clonal complexes (CC 3 and CC 13, Table 1) of the typical form. After excluding cases with excessive control mortality, we obtained enough replicates to quantify the virulence of 16 out of the 17 isolates tested. The virulence of these isolates in terms of honey bee brood mortality *in vitro* varied greatly (Figure 1). Three Swiss isolates (CH 21.1, CH 49.3 and CH 60) were highly virulent, causing over 80% mortality in infected brood up to the imago stage (Table 1, Table. S2, Figure. S3). These isolates caused significant mortality compared to the controls (Figure 1; pairwise log-rank tests, Bonferroni-Holm corrected, p < 0.001, Table. S1, Figure. S3). Eight isolates (FR 27.1, CH 119, CH 54.1, CH 46.1, CH 45.1, CH 40.2, NO 764–5B, NO 765–6B) had low to intermediate virulence, causing a brood mortality of 15 to 55%, which was significantly higher than that of the controls (Figure 1; pairwise log-rank tests, Bonferroni-Holm corrected, p < 0.001, Table. S1 and S2, Figure. S3). The mortality of the brood infected with each of the remaining five isolates (UK 36.1, UK 31.1, CH MeplS1, CH 82, IT 1.3) was not significantly higher than that of the non-infected control brood. These isolates were therefore categorized as avirulent (Figure 1; pairwise log-rank tests, Bonferroni-Holm corrected, n.s., Table. S1 and S2, Figure. S3).

**Table 1.** *Melissococcus plutonius* isolates with their collection location, year of isolation, multilocus sequence type and clonal complex. The presence of the melissotoxin A gene was assessed with a PCR screening test [39]. The post-infection mortality rate is expressed as a Henderson-Tilton corrected percentage. Avirulent isolates did not cause significant mortality compared to non-infected controls (Tab. S1) and Henderson-Tilton corrected mortality rates are inferior to 10%. Low to intermediate virulence degrees correspond to mortality rates in the range of 15–55%. High virulent isolates cause mortality rates above 80% (see Fig. S3).

Isolate code	Collection location (city, region and/or country)	Year	GenBank accession number	Sequence type (clonal complex)	Generation time ± SD [h]	Presence of melissotoxin Agene	Mortality	Virulence degree
UK 36.1	Somerset, England	2006	NA	ST 3 (3)	3.46 ± 0.98	NEGATIVE	1.24%	avirulent
UK 31.1	Norfolk, England	2006	NA	ST 13 (13)	3.83 ± 0.16	NEGATIVE	4.67%	avirulent
CH MeplS1^1^	Graubünden, Switzerland	2007	GCA_000747585.1	ST 3 (3)	3.49 ± 0.62	NEGATIVE	5.88%	avirulent
CH 82	Bern, Switzerland	2007	GCA_001047595.1	ST 32 (13)	5.40 ± 3.99	NEGATIVE	6.80%	avirulent
IT 1.3	Turin, Italy	2006	NA	ST 3 (3)	5.07 ± 1.42	NEGATIVE	7.43%	avirulent
FR 27.1	Gard, France	2006	NA	ST 20 (13)	4.28 ± 0.95	NEGATIVE	16.46%	low-intermediate
CH 90	Fribourg, Switzerland	2006	GCA_001047445.1	ST13 (13)	5.53 ± 2.77	NEGATIVE	22.55%	low-intermediate
CH 119	Bern, Switzerland	2007	GCA_001047515.1	ST 20 (13)	4.70 ± 1.25	NEGATIVE	28.28%	low-intermediate
CH 54.1	St. Gallen, Switzerland	2007	NA	ST 35 (13)	5.22 ± 1.42	NEGATIVE	32.82%	low-intermediate
CH 46.1	Zürich, Switzerland	2006	NA	ST 7 (3)	4.19 ± 2.30	NEGATIVE	39.44%	low-intermediate
CH 45.1	St. Gallen, Switzerland	2007	NA	ST 3 (3)	3.63 ± 1.02	NEGATIVE	42.93%	low-intermediate
CH 40.2	Zürich, Switzerland	2007	NA	ST 35 (13)	2.82 ± 0.70	NEGATIVE	43.97%	low-intermediate
NO 764–5B	Norway	2011	GCA_001047465.1	ST 3 (3)	4.52 ± 1.21	NEGATIVE	48.74%	low-intermediate
NO 765–6B	Norway	2011	GCA_001047435.1	ST 3 (3)	4.79 ± 1.26	NEGATIVE	53.50%	low-intermediate
CH 49.3	Graubünden, Switzerland	2007	GCA_001047395.1	ST 3 (3)	5.05 ± 3.98	POSITIVE	79.98%	high
CH 21.1	Bern, Switzerland	2006	GCA_001047455.1	ST 7 (3)	4.55 ± 0.80	POSITIVE	84.85%	high
CH 60	Bern, Switzerland	2007	GCA_001047545.1	ST 7 (3)	4.39 ± 1.49	POSITIVE	91.88%	high

^1^CH MeplS1 derives from re-cultivation of *M. plutonius* isolate CH 49.3 in the laboratory, and lost plasmid pMP19 including *melissotoxin A* [39].

### *Mortality caused by* M. plutonius, P. alvei *and co-infection*

**Figure 2.**
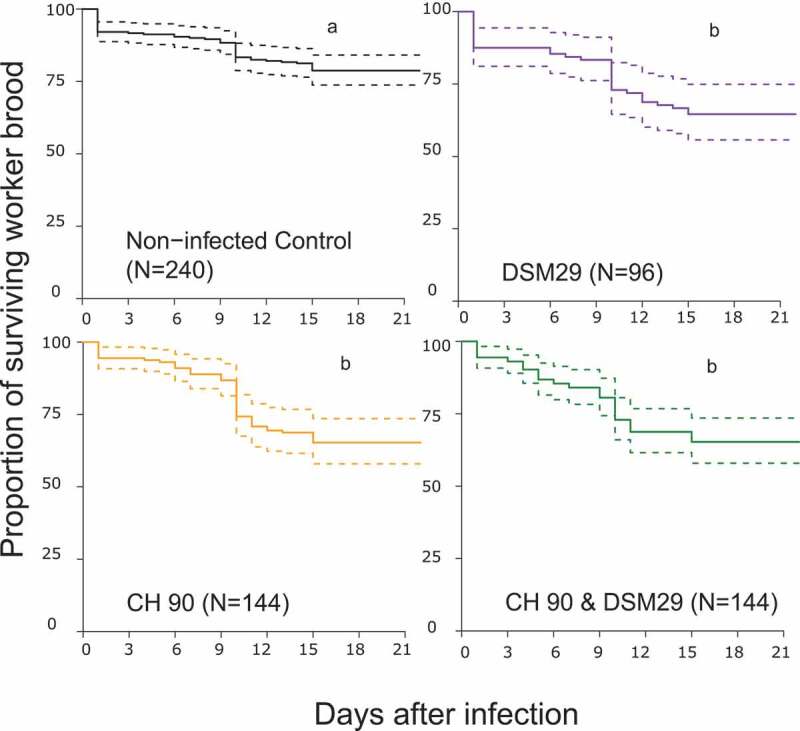
Survival of *in vitro* reared honey bees exposed to *M. plutoniu*s isolate CH 90, orange curve) or to *P. alvei* reference strain DSM29 (purple curve) only, or after co-infection with *M. plutonius* CH 90 and *P. alvei* DSM29 (green curve). The black curve indicates the survival of non-infected controls. Dashed lines represent 95% confidence intervals and N designates the number of larvae tested. Significant differences in the survival of brood due to treatments are indicated by different letters (pairwise log-rank tests, Bonferroni-Holm corrected, p < 0.01).

The survival of the honey bee brood after a single infection with *M. plutonius* CH 90 (Mean Henderson-Tilton corrected mortality 22.6%, Tab. S2) was significantly lower than the survival of the non-infected control brood (Figure 2, Table. S4; pairwise log-rank tests, Bonferroni-Holm corrected p = 0.006). Single infection with *P. alvei* DSM29 (Mean Henderson-Tilton corrected mortality 8.8%, Tab. S2) also caused significant mortality within the brood (Figure 2, Table. S4; pairwise log-rank tests, Bonferroni-Holm corrected p = 0.007), as did co-infection with *M. plutonius* CH 90 and *P. alvei* DSM29 (Figure 2, Table. S2 and S4; pairwise log-rank tests, Bonferroni-Holm corrected p = 0.004; Mean Henderson-Tilton corrected mortality 23.1%). However, no significant increase in mortality was observed after co-infection with *M. plutonius* and *P. alvei* compared to single infections with one bacterial species alone (Figure 2 Tab. S4, pairwise log-rank tests, Bonferroni-Holm corrected p > 0.01).

### *Growth dynamics and virulence of* M. plutonius

**Figure 3.**
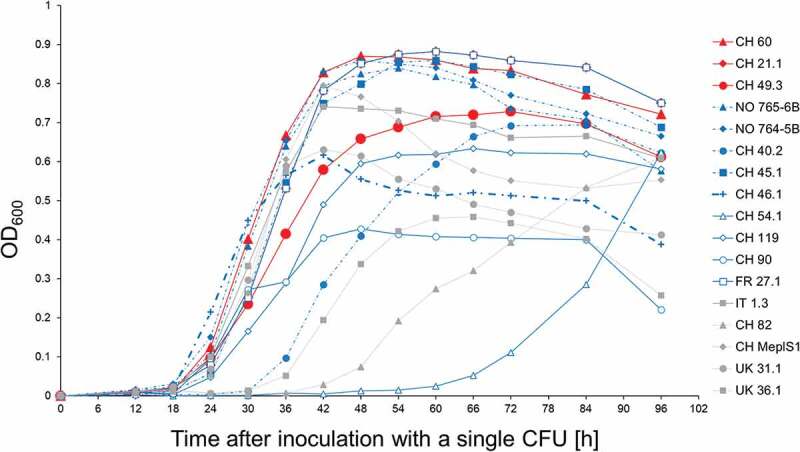
Growth curves of 17 *M. plutonius* isolates in artificial medium. Red, blue and gray curves correspond to high, low-intermediate virulence and avirulent isolates, respectively. See Tab. S5 for mean OD_600_-values and SD and table S6 for statistical differences in growth dynamics between isolates.

The *M. plutonius* isolates varied in their *in vitro* growth dynamics (Figure 3). Some bacterial isolates multiplied rapidly to high cell densities, while others multiplied slowly and reached low final cell densities in liquid basal medium at the end of the observation period. Other strains showed mixed features, multiplying rapidly to low final cell densities and inversely. Four isolates of low to intermediate virulence (FR 27.1, CH 45.1, NO 764–5B and NO 765–6B) and one avirulent isolate (IT 1.3) showed no significantly different growth pattern as isolates of high virulence (CH 49.3, CH 60 and CH 21.1; Figure 3, Tables. S5 and S6). OD_600_ of most isolates decreased abruptly after 84 h, indicating bacterial mortality. We thus considered this time point as indicative of final density reached. Four isolates of low to intermediate virulence (FR 27.1, CH 45.1, NO 764–5B and NO 765–6B) reached final densities as high as those measured in high virulence isolates (CH 49.3, CH 21.1 and CH 60; Figure 3, Table. S5). *M. plutonius* isolates causing over 40% mortality (corrected through a Henderson-Tilton calculation) in the *in vitro* larval infection assay, also reached high final densities in basal medium with OD_600_ values at 0.7 or above, and five out of the seven most virulent isolates reached OD_600_ values above 0.8 (Tab. S5).

### Presence of melissotoxin A gene

The three highly virulent *M. plutonius* isolates CH 21.1, CH 49.3 and CH 60 were positive for the gene encoding melissotoxin A in PCR screening, while all other isolates were negative (Table 1, S8).

### Relative contribution of sequence type, clonal complex, growth rate, final cell density and melissotoxin A to the virulence of M. plutonius

The multiple regression model, including sequence type, clonal complex, generation time, final cell densities and the presence of melissotoxin A gene, explained a significant part of brood mortality (R^2^ = 0.806, F_5,15_ = 7.483, p < 0.005; Tab S7). While the presence of the plasmid detected through the amplification of a 1.36 kbp-fragment of a putative toxin gene significantly predicted brood mortality (B = 45.802, β = 0.617, p < 0.007), neither sequence type (B = 0.757, β = 0.270, p = 0.422), nor clonal complex (B = −2.419, β = −0.384, p = 0.277), nor generation time (B = −2.952, β = −0.068, p = 0.671), nor cell density reached at 84 h (B = 74.226, β = 0.342, p = 0.068) was significantly associated with brood mortality. The model did not show any correlation between the mortality rate induced in the honey bee brood (N = 15, Kendall’s τ = 0.175, p = 0.266; Tab. S7) and the generation time of the tested *M. plutonius* isolates during their exponential growth phase in artificial medium, which ranged from 3 to 6 h (Table 1). In contrast, final cell density (at 84 h) was positively and significantly correlated with mortality (N = 15, Kendall’s τ = 0.641, p < 0.005, Tab. S7).

## Discussion

### *Virulence varies among* M. plutonius *isolates*

Mortality of the honey bee brood caused by the *M. plutonius* isolates tested varied greatly. The variation in virulence among the 16 isolates was independent of their multi-locus sequence type and clonal complex affiliation. Variation in virulence among strains of the so called “atypical” *M. plutonius* belonging to different clonal complexes has recently been documented [41]. Our results indicate that variation in virulence also occurs in clonal complexes belonging to the “typical” strains of *M. plutonius* as observed in [42]. Mortality rates also varied among replicates of *in vitro* infection tests using the same *M. plutonius* isolate, but larvae of different colonies. This is in line with the results from two recent studies [41,42] and may be due to variation in host susceptibility. Although all but one isolate (CH MeplS1) tested in this study came from honey bee colonies with explicit symptoms of an acute EFB infection, four isolates (UK 36.1, UK 31.1, CH 82 and IT 1.3) did not cause elevated larval mortality *in vitro*, and were therefore ranked as avirulent. It is possible that this discrepancy is a methodological artifact due to the loss of the plasmid carrying the putative virulence factor during cultivation on artificial media. Although the tested isolates were subjected to the same laboratory procedures, random loss of the plasmid cannot be excluded. Alternatively, negative effects on the development or physiology of the larvae not detected in our assay could have triggered increased removal by adult hygienic workers, generating the typical spotty brood pattern in EFB-diseased colonies. It is also possible that bacterial isolates less virulent at individual level are highly virulent at colony level, and *vice versa*, as was hypothesized for *Paenibacillus larvae* [63]. Larvae infected with low virulence isolates might be removed slower than larvae affected by high virulence isolates. A low removal rate could facilitate the production and spread of the pathogenic bacteria within the colony. It is highly likely that the *in vivo* virulence of *M. plutonius* at colony level is modulated by the effect of the social immunity of the colony [64], as proposed for *P. larvae* [63,65] and be different from virulence quantified *in vitro*.

### *No evidence that co-infection with* P. alvei *increases mortality*

Our results indicate that the presence of the secondary agent *P. alvei* is not required to induce brood mortality. We observed high larval mortality after single infection with some of the *M. plutonius* isolates, in line with former studies [34,41,42,62]. To our knowledge, this is the first report on single infections with *P. alvei* in an *in vitro* infection assay. In this study, single infection with *P. alvei* strain DSM29 caused significant mortality to the honey bee brood. In contrast, co-infection with *M. plutonius* did not increase brood mortality beyond the effect of single infections with the tested *M. plutonius* isolate (CH 90). These results are similar to those of Lewkowski and Erler, 2018 [42], but differ from those of Giersch *et al*., 2010 [47], probably because of methodological differences (*e.g*., different infection time, higher concentration of *P. alvei* used in their study). To better understand the role of secondary agents, other bacteria associated with EFB should be tested singly and in co-infections with a larger set of *M. plutonius* isolates *in vitro* [42] as well as *in vivo* in honey bee colonies.

### *Relationship between* M. plutonius *growth dynamics and virulence*

The growth dynamics of *M. plutonius* isolates in the culture medium were highly variable. The bacterial generation time in the artificial medium did not predict mortality in the honey bee brood. The final bacterial density may play a more important role in determining pathogenicity (*sensu* [66]) than the generation time. All isolates defined as being of intermediate or high virulence reached high final densities in the culture medium, and isolates with lower final densities caused low mortality. This pattern obtained *in vitro* suggests that strains must reach a damage threshold to induce high mortality. Although the growth dynamics of *M. plutonius* isolates in culture medium as observed in this study may be different from that in honey bee larvae, the hypothesis that a high bacterial load is a prerequisite for high virulence is supported by the occurrence of a positive correlation between bacterial numbers and virulence *in vivo* [41,62]. However, some low virulence isolates reached high final densities in the culture medium at 84 h, suggesting that factors other than the number of *M. plutonius* bacteria are involved in causing high mortality in honey bee brood.

### Role of melissotoxin A in virulence

The melissotoxin A gene was restricted to three highly virulent Swiss isolates (CH 21.1, CH 49.3 and CH 60) and the presence of this gene was a significant predictor of honey bee brood mortality (Tab. S5). In our experiment, the bacterial isolate CH MeplS1 was avirulent. This isolate originated from a bacterial culture of the highly virulent isolate CH 49.3 and lost plasmid pMP19 (19.4 kbp; GenBank: JSBA01000009.1) after repeated sub-cultivation [39]. Plasmid pMP19 encodes melissotoxin A (GenBank: KMT29105) and another putative virulence factor, the extracellular matrix-binding protein [39]. While the additional loss of genome-encoded virulence factors in CH Mepl S1 cannot be excluded, the simultaneous loss of pMP19 and virulence suggests a causal link.

A further piece of evidence for the possible implication of the melissotoxin A gene in virulence is that it is expressed by *M. plutonius* during infection *in vivo* [39,62]. This gene shares a high sequence similarity with the epsilon toxins of the ETX/MTX2 family (pfam03318 of *Clostridium perfringens*, 33% amino acid sequence identity [39] and of *Bacillus pumilus* with 48% sequence identity, 90% coverage and an Expect (E)-value of 7e^-73^, NCBI query; https://www.ncbi.nlm.nih.gov/). Epsilon toxins change the cell permeability for ions by forming large membrane pores, causing cell death and edema in animal models [67]. It was recently suggested that a protein of the ETX/MT2 family is an important virulence factor in two subtypes of *P. larvae*, the causative pathogen of American foulbrood [68]. It is thus plausible that melissotoxin A increases the virulence of *M. plutonius* by corrupting the cells of the larva’s digestive tract.

The melissotoxin A gene is situated on plasmid pMP19 [39]. Plasmids can easily be exchanged between individual bacterial cells within and between species [69]. The plasmid pMP19 found in certain *M. plutonius* strains, including atypical isolates from Japan [40,62], may originate from other bacterial invaders co-existing with *M. plutonius* in the intestinal tract of honey bee larvae. The fact that the toxin gene is located on a mobile genetic element can explain the absence of association between brood mortality and the sequence type or clonal complex of the isolates used in this study.

The conventional explanation for the negative effect of *M. plutonius* is a competition for nutrients in the gut [25]. Although not mutually exclusive, our finding that a toxin-carrying mobile genetic element could confer high pathogenicity to certain *M. plutonius* isolates suggests a direct detrimental effect on gut cells. This mode of action is in line with the findings of McKee *et al*., 2004 [46] and with evidence that *M. plutonius* still caused high larval mortality in *in vitro* experiments where food was given in excess [34,41,42,46,47,60–62].

Further experiments will be needed to demonstrate the direct role of melissotoxin A in the pathogenicity of *M. plutonius*. Such experiments include producing genetically modified strains of *M. plutonius*, for example by removing plasmid pMP19 from highly virulent strains, knocking-out melissotoxin A gene on pMP19 (pMP19Δmelissotoxin A gene), transforming wildtype pMP19 and pMP19Δmelissotoxin A gene into low virulent isolates of *M. plutonius*, as well as producing the putative toxin in the laboratory. The impact of mutant strains or the synthesized toxin on honey bee larvae could then be directly tested *in vitro*. Curing pMP19 from Japanese *M. plutonius* isolates belonging to CC 3 resulted in a loss of virulence [62], supporting our hypothesis for the role of the melissotoxin A gene. However, transformation experiments aimed at introducing the plasmid back in the cured isolates did not result in recuperation of virulence [62]. A reduced growth of these isolates due to the transformation protocol was provided as an explanation [62], in line with our suggestion that rapid multiplication is also required to produce highly virulent phenotypes. The role of the putative virulence gene on pMP19 could not be confirmed in a Japanese isolate of CC 13 [62]. A reduced growth of this isolate could also explain its low virulence despite the gene’s presence.

### Is the recent emergence of EFB due to highly virulent strains?

The question then arises as to whether the recent emergence of EFB is linked to the occurrence of bacterial strains of particularly high virulence at the colony level. Virulence trade-off models predict that the virulence of a pathogen is inversely correlated to its transmission, because excessive virulence causes early host death, hindering the spread of the pathogen amongst the population [70–73]. However, human-induced changes in the rate of horizontal transmission may have altered the adaptive compromise between virulence and transmission in *M. plutonius* [17,74,75]. Increased contact between honey bee colonies due to human management may allow for the spread of highly virulent strains unable to spread to new hosts under more natural settings.

More extensive field screening of the prevalence of different *M. plutonius* sequence types and of the plasmid carrying the melissotoxin A gene will reveal if highly virulent strains of *M. plutonius* arose by acquiring this toxin-carrying mobile genetic element. This will also contribute to a better understanding of the origin of recent epidemic outbreaks of EFB. If a link between virulence and disease outbreaks in the field can be established, strain-specific, virulence-based control methods can be promising avenues to better control the spread of EFB [29,30]. The melissotoxin A gene could provide a putative marker for such specific control methods.

### Conclusion

Infection with *M. plutonius* alone caused European foulbrood symptoms in honey bee brood reared *in vitro*. Bee mortality varied substantially among 16 investigated *M. plutonius* isolates, independently of their sequence type. Mortality did not increase when larvae were infected with the potential secondary agent *P. alvei* in combination with a *M. plutonius* isolate of low to intermediate virulence. Variation in the virulence of *M. plutonius* isolates was not significantly linked to growth dynamics. A high final density in the culture medium was reached by highly virulent isolates, but was not sufficient to explain the high mortality caused by these *M. plutonius* isolates. High virulence was associated with the presence of a plasmid carrying the putative toxin melissotoxin A, suggesting that this mobile genetic element is a major factor in the virulence of *M. plutonius*. These new insights into the pathogenicity of a poorly understood, but important honey bee disease may serve as a basis for the development of more sustainable control methods.

## Experimental Procedures

### *Origin and culture of* M. plutonius *isolates*

Diseased larvae were collected from European foulbrood (EFB) outbreaks in several European regions (Table 1) and sent to our laboratory for the purpose of *M. plutonius* isolation. The only exception was the Norwegian samples, which were processed elsewhere. *Melissococcus plutonius* was isolated from smears of diseased larvae on basal medium. The medium contained 1% yeast extract, 1% glucose, 1% saccharose, 0.04% L-cysteine and 0.1 M KH_2_PO_4_ in distilled water, with a pH adjusted to 6.7 with 5 M KOH. The medium was solidified with 18 g agar/liter and autoclaved at 121°C for 18 min [58,76]. After incubation for four days at 36°C under anaerobic conditions (GENbox anaer, bioMérieux), individual bacterial colonies identified as *M. plutonius* based on colony morphology were picked from the Petri dishes and inoculated in a liquid basal medium or on plates. The plates were incubated anaerobically for another four days at 36°C. Isolate stock solutions were supplemented with 15% glycerol and stored at −80°C until further use.

### *PCR identification of* M. plutonius *and detection of the gene encoding melissotoxin A*

Isolates were confirmed as *M. plutonius* by PCR as described by Govan *et al*., 1998 [74]. The strains were further identified as either “typical” or “atypical” following the protocol described in Arai *et al*., 2014 [77]. The multi-locus sequence type and clonal complex of each isolate were assessed as described in Haynes *et al*., 2013 [36]. We screened for the presence of melissotoxin A gene (GenBank: KMT29105) [39] using specific primers (tox_MEPL_for: 5ʹ-GCTCAAGCAGCAACTTTTACG-3ʹ and tox_MEPL_rev: 5ʹ-TTCCCCTGGTATTACTTGTAGATG-3ʹ; fragment size approx. 1.36 kbp) in a conventional PCR reaction using KAPA2 G Fast DNA Polymerase (KAPA Biosystems). DNA was extracted with the NucleoSpin Tissue kit from Macherey-Nagel according to the manufacturer’s instructions, the DNA-concentration was measured with NanoDrop® ND-1000 spectrophotometer (NanoDrop, Thermo Fischer Scientific, USA) and each DNA-extract diluted to 5 ng DNA per µl. Each reaction in the PCR consisted of 2 µl template DNA-extract, 13 µl KAPA2 G fast ready mix (2x), 1 µl forward primer (10 µM), 1 µl reverse primer (10 µM), and 8 µl water added to a total reaction volume of 25 µl. The PCR started with an initial denaturation step at 95°C for 3 min, followed by 40 cycles of denaturation at 95°C for 15 s, annealing at 58°C for 15 s and DNA extension at 72°C for 20 s, and a final extension step at 72°C for 2 min (TProfessional Basic Thermocycler, Biometra). PCR products were visualized under UV light after staining the 1.5% agarose gel with GelRed™ (Biotium).

### *Infection assays of honey bee larvae reared* in vitro

Honey bee brood originating from healthy, queenright colonies was used in the infection assays. Same aged larvae were obtained by confining queens to empty combs for the purpose of egg-laying, using excluder cages. After 36 hours, the queens were removed from the cages for three days, until first instar larvae hatched. First instar larvae were grafted individually in plastic queen starter cells (Nicoplast™) that had been sterilized in 70% ethanol for 30 min. The plastic cells were placed in the cells of 48-well tissue culture plates, on top of a piece of wet dental roll imbibed with 15.5% glycerol in 0.4% methyl benzethonium chloride to prevent unwanted microorganism growth. The larvae were reared *in vitro*, according to standard methods [59]. In brief, the culture plates were placed in a hermetic desiccator containing a dish filled with saturated K_2_SO_4_ solution, which ensured a relative humidity of 95% needed to prevent dehydration of larvae. The desiccator containing the larvae was placed into an incubator at 34.5°C for the first six days of larval development. At day 7, when the larvae defecate and start to pupate, the plates were moved into a desiccator containing a dish filled with saturated NaCl solution to ensure an optimal relative humidity of 75%. The larvae were fed daily with pre-warmed diet (34.5°C), according to the following feeding program: on the day of grafting, larvae were provided with 10 µl of diet A (1.2 g glucose, 1.2 g fructose, 0.2 g yeast extract and 8.4 g pure water, filter sterilized (0.2 µm) and mixed with 10 g pure royal jelly). On day 3, larvae were fed 20 µl of diet B (1.5 g glucose, 1.5 g fructose, 0.3 g yeast extract and 8 g pure water, filter sterilized (0.2 µm) and mixed with 10 g pure royal jelly). On days 4, 5 and 6, larvae were fed with 30, 40 and 50 µl of diet C, respectively. Diet C consisted of 1.8 g glucose, 1.8 g fructose, 0.4 g yeast extract and 7.45 g pure water, filter sterilized (0.2 µm) and mixed with 10 g pure royal jelly [59,78]. The royal jelly was obtained from healthy colonies and stored at −20°C.

For *in vitro* infection experiments, *M. plutonius* isolates were cultivated in a liquid basal medium at 36°C under anaerobic conditions for four days. In order to standardize the number of bacteria administered in the infection assays, we determined the concentration of viable bacterial cells using colony forming units (CFUs) counting. The CFUs from a serial dilution spread on basal medium agar plates were counted after four days of incubation at 36°C under anaerobic conditions [76]. During this time and before administration, bacterial cultures were stored at 4°C.

*Paenibacillus alvei* type strain DSM29 was obtained from the German Culture Collection (DSMZ, Braunschweig, Germany) and cultivated on casein-peptone agar plates under aerobic conditions at 30°C for three weeks, until spores formed. Spores were harvested from agar plates by scraping them off the plate and suspending them in saline (0.9% NaCl in distilled water). The spores were washed once with saline, diluted in saline and heat treated for 5 minutes at 90°C. The total *P. alvei* spore concentration was determined via 10-fold dilutions plated on casein-peptone agar plates and CFU counting [79]. Spore suspensions were stored at 4°C.

Infection with *M. plutonius* was induced by administering a droplet of 10 µl diet A spiked with 10^7^ CFU ml^−1^ of the respective *M. plutonius* isolate (1:9 *M. plutonius* inoculum-diet mix) on day 1, within two hours of grafting. Hence, each larva was fed 10^5^ CFU of *M. plutonius*. Larvae were infected with *P. alvei* on day 1 by receiving 10 µl of diet A spiked with 10^5^ spores of *P. alvei* per ml of saline (i.e. 10^3^ *P. alvei* spores per larva). Co-infections were performed by feeding 10 µl diet A spiked with a mix of 10^5^ *P. alvei* DSM29 spores ml^−1^ and 10^7^ CFU ml^−1^ of *M. plutonius* isolate CH 90. This isolate was selected for co-infection assays for its relatively low virulence, which facilitates the detection of a putative increased mortality due to *P. alvei*. Non-infected control larvae received 10 µl of diet A mixed with sterile saline. We administered a lower number of *P. alvei* spores per larva (10^3^) than in other studies (6 x 10^4^; [42,47]). This lower dose is closer to the one eliciting American foulbrood symptoms in infections by the congeneric bacteria *P. larvae* (20 spores/larva; [80]), and comparable to the number of *P. alvei* spores larvae were exposed to in a recent study (8.3 x 10^3^; [42]).

Honey bee larvae were subjected to single infections with *M. plutonius*, single infections with *P. alvei* and co-infections with *M. plutonius* and *P. alvei*. We monitored brood survival and compared brood mortality caused by the isolates, alone or in combination. The status of each larva (dead or alive) was recorded every 24 h by observing it under a microscope. Larvae or pupae without signs of respiration or reaction to mechanical stimulus were recorded as dead and removed from the plates [78]. In contrast to other studies [34,41,42,46,47], we monitored brood survival until completion of development (*i.e*. until imaginal stage) to take into account the whole brood developmental period to assess its mortality. For each strain or combination of strains, 2–6 replicates were performed, each with 24–84 larvae produced by 2–8 queens (Tab. S2).

### *Growth dynamics of* M. plutonius *isolates*

For each *M. plutonius* isolate used in the infections assays (17 isolates), ten replicates (two runs with five replicates each) of 20 ml liquid basal medium were inoculated with a single *M. plutonius* colony pre-cultured on an agar plate for three days and incubated under anaerobic conditions at 36°C. Bacterial growth was monitored by measuring optical density at 600 nm (OD_600_) with a spectrophotometer (DR/2000, HACH), every 6 h until 96 h past inoculation [81]. OD_600_ was averaged over the ten replicates. Based on this data, we calculated the generation time and compared the growth dynamics of the isolates (see below).

### Statistics

Survival differences in honey bee brood after experimental infection with *M. plutonius* or *P. alvei*, or with a combination of *M. plutonius* and *P. alvei*, were illustrated with Kaplan-Meier survival curves with 95% confidence intervals [82]. Differences in survival between hosts infected by various isolates, and between the latter and uninfected controls were tested using pairwise log-rank tests (Mantel-Haenszel test [83]) and adopting a significance level α of 0.05, corrected by a Bonferroni-Holm procedure for multiple comparisons [84]. We calculated the Henderson-Tilton corrected mortality rate of individuals dead until day 21 of development, when the imago stage is reached, as follows: mortality ratio = 1- ((number of live test bees after treatment * number of live control bees after treatment^-1^) * (number of live control bees before treatment * number of live bees before treatment^-1^)) [85], and state the resulting mortality rate caused by each tested *M. plutonius* isolate in Table 1.

We used the R package growthrates [86] to compute the growth constant k for each isolate, by fitting segments of linear models to the log-transformed OD_600_ values during the exponential growth phase [81]. The generation time (g) of each isolate was then calculated [87] as g = ln (2)/k. As recommended by Hall *et al*., 2013 [81], we verified the fit to the exponential phase generated by the algorithm implemented in Petzoldt, 2016 [86]. For isolate CH 54.1, the algorithm did not identify the exponential phase correctly, due to a long and irregular lag phase. We obtained a corrected dataset by excluding the first six points for this isolate well ahead of the exponential phase, which led to correct fitting. The overall correlation between growth rate and mortality, as well as the results of the regression model, were similar between the corrected dataset, the original dataset, and after excluding this isolate from the dataset (Tab. S7). We chose to present conservative results of the regression model, based on the dataset excluding CH 54.1.

Generation time only considers the exponential phase of the bacterial growth cycle. To analyze the growth dynamics over the entire experiment (96 h), we used permutation tests with 100,000 iterations to conduct pairwise comparisons between growth curves [88,89]. These statistical analyses were done in R (R Foundation for Statistical Computing, Vienna, Austria) with the statmod package [90].

A multiple regression analysis [91] was conducted to examine the relationship between the Henderson-Tilton corrected mortality and the potential virulence factors multi-locus sequence type, clonal complex, generation time, final density (density reached at 84 h) and presence of melissotoxin A gene. For this model, we used SPSS Statistics, version 21 (IBM Corp.).

## Supplementary Material

Supplemental Material
