# Supplementary material for: Putative determinants of virulence in Melissococcus plutonius, the bacterial agent causing European foulbrood in honey bees
Source: Virulence. 2020 May 26;11(1):554–67. doi: 10.1080/21505594.2020.1768338 (PMC7567439; doi:10.1080/21505594.2020.1768338)
Supplement: Supplemental Material [file KVIR_A_1768338_SM4380.docx]

# **SUPPLEMENTARY MATERIAL**

**Table S1.** Results matrix of Kaplan-Meier survival curves comparisons after experimental infection of honey bee larvae with *M. plutonius* isolates. Survival of non-infected control larvae (C) and larvae infected with 16 *M. plutonius* isolates was compared using pairwise log rank tests (Mantel-Haenszel-test), of which the p-values are reported in the table. Significant differences after Bonferroni-Holm correction method are shown in bold.

**Table S2.** Henderson-Tilton corrected mortality rates in percent with standard deviation (SD), minimal (Min) and maximal (Max) value, the number of runs (Runs), number of larvae (N) and number of mother honey bee queens (Queens) used per treatment. Control mortality in percent (Mean ± SD) is given for single (N= 462, N_Queens_= 10, N_Runs_= 10) and co-infection assays (N= 240, N_Queens_= 8, N_Runs_= 5).


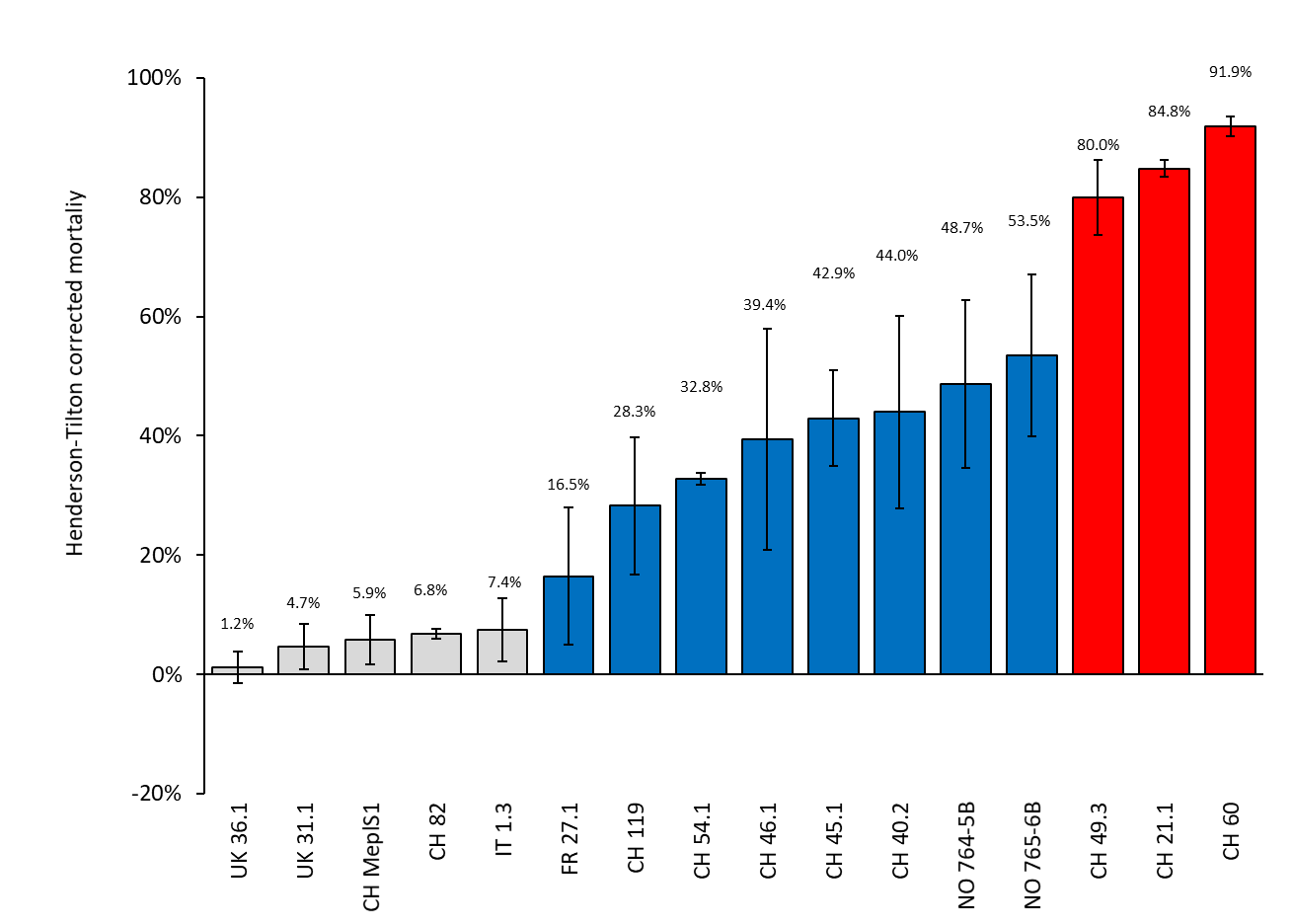


**Fig. S3.** Henderson-Tilton corrected honey bee brood mortality rates (mean±SE) after infection with 16 *M. plutonius* isolates. Isolates are ordered by increasing virulence. Blue and red bars correspond to low to intermediate and high virulence isolates, respectively. Grey bars represent avirulent *M. plutonius* isolates The three categories were determined based on statistical comparisons of virulence with controls (mortality of brood infected with avirulent strains was not significantly different from that of non-infected brood) and on large differences in virulence range (i.e., between NO 765-6B and CH 49.3).


**Table S4.** Results matrix of Kaplan-Meier survival curves comparisons after experimental co-infection of honey bee larvae with *M. plutonius* and *P. alvei*. The Survival of non-infected control larvae (C), of larvae infected with *P. alvei* isolate DSM29, of larvae infected with *M. plutonius* strain CH 90, and of larvae co-infected with *M. plutonius* CH 90 and *P. alvei* DSM 29, was compared using pairwise log rank tests (Mantel-Haenszel-test) and the p-values for these tests are reported in the table. Significant differences after Holm-Bonferroni correction method are bold.

**Table S5**. Mean OD_600_-values ± SD as measure for growth of 17 *M. plutonius* isolates in liquid basal medium over a time period of 96h. Final cell densities are defined as an OD_600_-value above 0.7 after 84 h.

**Table S6**. Comparisons between growth dynamics of 17 *M. plutonius* isolates. Optical density of culture medium at 600nm every six hours was compared pairwise with a permutation test with 100,000 iterations. P-values are reported in the table, and differences that are significant after Holm-Bonferroni correction method are shown in bold.

**Table S7.** Results of the statistical analyses of growth constant k based on original dataset, corrected dataset (first six data points of the lag phase of isolate CH 54.1 excluded due to due to a long and irregular lag phase observed in this isolate, see data analysis section of methods) and original dataset without isolate CH 54.1.


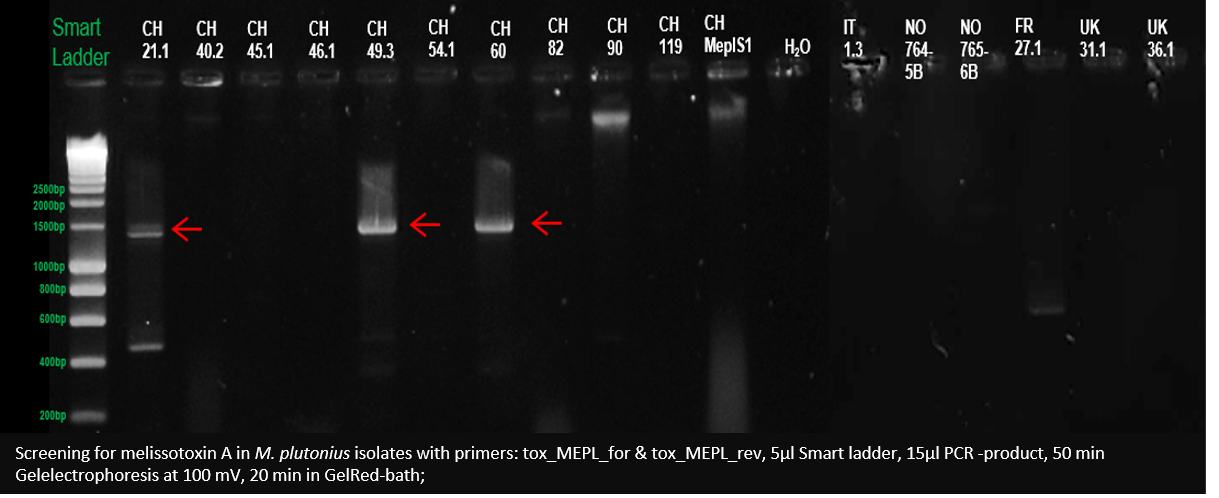


**S8.** Picture of the agarose gel showing the results of the PCR screening for melissotoxin A gene in *M. plutonius* isolates used in this study.
